# Poor prognosis of retroperitoneal mixed extragonadal germ cell tumors in an HIV-infected man with severe immunosuppression and bilateral cryptorchidism: a case report

**DOI:** 10.1186/s12885-019-5456-0

**Published:** 2019-03-18

**Authors:** Ruili Li, Hongjun Li

**Affiliations:** Department of Radiology, Beijing Youan Hospital, Capital Medical University, No. 8, Xi Tou Tiao, Youanmen Wai, Fengtai District, Beijing, 100069 China

**Keywords:** Nonseminomatous germ cell tumor, Extragonadal, HIV, Immunosuppression, Poor prognosis

## Abstract

**Background:**

Nonseminomatous germ cell tumors (NSGCTs) represent one of the main groups of germ cell tumors (GCTs), and they have a more invasive course than seminomatous GCTs. Human immunodeficiency virus (HIV) positivity is considered to be a risk factor for testicular seminoma patients, but reports about HIV-infected individuals with NSGCTs are rare.

**Case presentation:**

We report a case of a retroperitoneal mixed extragonadal germ cell tumor in an HIV-infected man who has been diagnosed with bilateral cryptorchidism since birth. A 30-year-old man presented with a large heterogeneously mixed echo mass located in the right lower abdomen according to an abdominal ultrasound; he was HIV-positive and had a low CD4 count of 70 cells/ml in the followed test, which suggested severe immunosuppression, and ultrasound-guided biopsy histology revealed a malignant yolk sac tumor of the testis. First, the patient received combination antiretroviral therapy; then, to relieve his symptoms, an exploratory laparotomy and retroperitoneal neoplasm resection under general anesthesia were performed for subsequent treatment. The postoperative histopathological examination indicated that the patient exhibited malignant mixed GCTs of the undescended testis that were composed predominantly of yolk sac tumors with foci of embryonal cell carcinoma and seminoma; It is a rare type in various GCTs, especially in HIV-infected patients. After the operation, the patient underwent computed tomography follow-up scans at 1 week and 2 weeks, and the results showed that the size of the right inguinal mass gradually increased, which suggested a poor outcome. To limit the growth of the tumors, right inguinal mass resection under local anesthesia was performed 17 days after the initial operation, and pathological examination revealed mixed GCT metastasis. Subsequently, the patient received salvage chemotherapy with a regimen of cisplatin, etoposide, and ifosfamide. Unfortunately, the patient died 1 week after the first cycle of chemotherapy because of severe immunosuppression, a low platelet count and cancer cachexia.

**Conclusions:**

Because of severe immunosuppression, the treatment of advanced extragonadal NSGCTs in an HIV-infected patient resulted in a poor prognosis. This outcome should be considered in further research, and appropriate management for achieving long-term survival needs to be established.

## Background

Germ cell tumors (GCTs) in males mainly develop in testicular tissue; 1–5% of GCTs occur in extragonadal sites and are defined as extragonadal germ cell tumors (EGGCTs) [1]. GCTs include seminomas (35–71%) and nonseminomatous germ cell tumors (NSGCTs) [2]. Cryptorchidism is a certain risk factor for testicular GCTs [3]. With the worldwide human immunodeficiency virus (HIV) pandemic, seminoma appears to be more common among HIV-infected subjects, but NSGCTs are rare [4]. The standardized treatment guidelines for non-HIV-infected GCT patients may not be applicable to HIV-infected patients, especially those with severe immunosuppression. Here, we describe a rare case of retroperitoneal mixed EGGCTs in an HIV-infected man with severe immunosuppression and bilateral cryptorchidism, and the clinical manifestations, treatment, and prognosis are reported.

## Case presentation

**Fig. 1:**
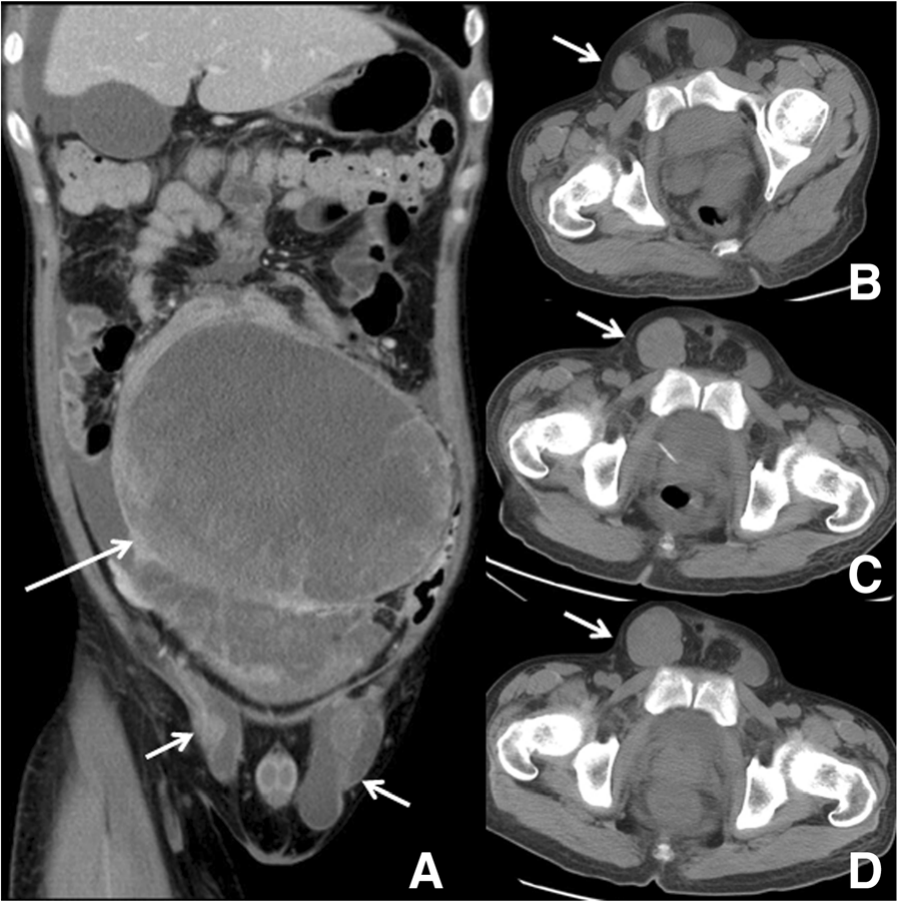
CT imaging findings of the case. **a** Coronal contrast-enhanced CT scan showed a retroperitoneal heterogeneous enhancement mass with multiple large necrotic areas (long arrow) and cystic-solid masses in the bilateral inguinal regions (short arrow). **b**-**d** The right inguinal region mass gradually increased (short arrow) (5 days, 18 days, 25 days after admission)

A 30-year-old man complained of a gradually enlarged mass in the right lower abdomen. The results of an abdominal ultrasound taken at the local hospital 3 months prior showed a heterogeneously mixed echo mass located in the right lower abdomen, and the size of the mass was approximately 8.6 cm × 7.3 cm. He had no family history of malignancy but had a history of bilateral undescended testis since birth. The local medical officer suspected a testicular tumor according to the history of cryptorchidism. At the same time, his rapid HIV-1 antibody test showed positive results, and the baseline CD4 count was 70 cells/ml (normal: 404–1612 cells/ml) upon further testing, which suggested severe immunosuppression. The patient initially received combination antiretroviral therapy (cART) but refused treatment for the abdominal mass. As the mass rapidly grew for 3 months, he came to our hospital for treatment of abdominal neoplasm. Upon examination, an immobile and nontender mass was visibly noticeable and palpable in the right lower abdomen. The bilateral testis was not visualized and could not be palpated. The patient had significantly elevated levels of alpha-fetoprotein (AFP), slightly elevated levels of beta-human chorionic gonadotropin (β-HCG), moderately decreased levels of hemoglobin and a low CD4 count (Table 1). Further evaluation revealed a low viral load, which was less than 40 copy/ml. Computed tomography (CT) images showed a large, lobulated, ill-defined heterogeneous retroperitoneal mass measuring 17 cm × 16 cm × 24 cm without fat or calcifications and with marked inhomogeneous enhancement due to the presence of necrotic-colliquative areas (Fig. 1a). The lesion displaced the bilateral lower ureters, resulting in bilateral hydronephrosis. The lesion also compressed the surrounding small intestine, with possible infiltrating signs. Around the mass, ascites was detected, but no enlarged lymph nodes were found. Cystic-solid masses (5 cm × 5 cm) were detected in the bilateral inguinal regions, and their density and enhanced characteristics were similar to those of the retroperitoneal neoplasm (Fig. 1a, b). A diagnosis of testicular tumors with bilateral inguinal region metastases was suspected. The patient underwent routine clinical staging and prognosis evaluations according to the results of radiological and laboratory examinations. Clinical staging and risk group categorization were classified as stage IIIC and poor prognosis using the American Joint Committee on Cancer staging system (AJCC) [5] and the International Germ Cell Cancer Collaboration Group (IGCCCG) prognostic scoring scheme (IGCCCG, 1997), respectively [6].

**Table 1:** The pre- and postoperative hematological and tumor parameters of the patient

	Pre	Post (2 days later)	Normal range
AFP (ng/ml)	34,222	8145	0–7
β-HCG (IU/L)	9.2	1.8	0–2
Hemoglobin (g/L)	77	64	130–175
CD4+ count (cells/ml)	86	78	404–1612

**Fig. 2:**
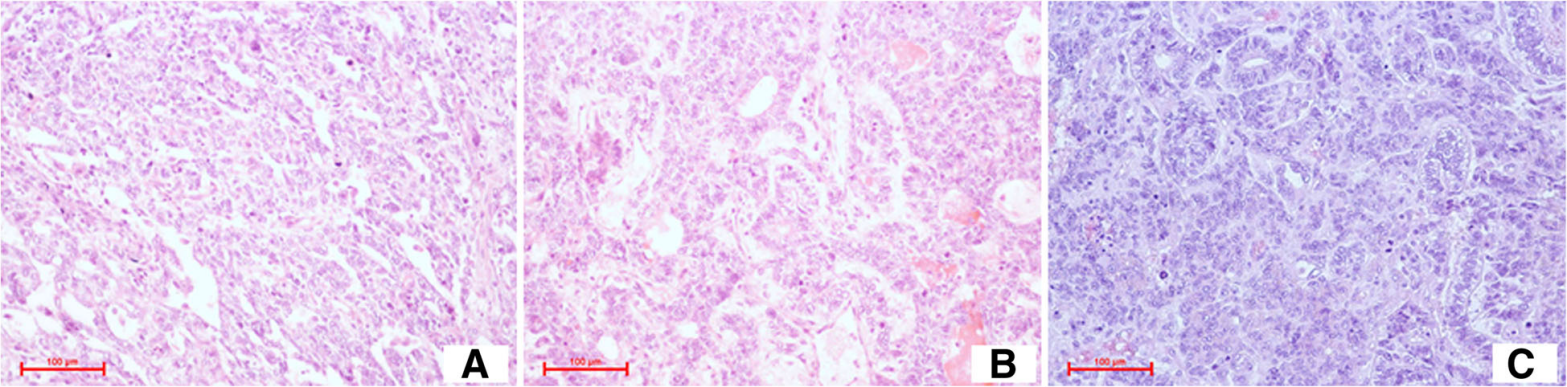
Pathology findings of the case. **a**, **b** Hematoxylin and eosin (H&E) staining (× 200). Embryonal carcinoma component and yolk sac tumor component (retroperitoneal mass). **c** H&E staining (× 200). Yolk sac tumor component (inguinal mass)

An ultrasound-guided biopsy was performed on the 5th day after admission, and histology revealed a malignant yolk sac tumor of the testis. Then, the patient underwent exploratory laparotomy, retroperitoneal neoplasm resection, partial ileum resection, ileal anastomosis, and double J ureteral stent implantation under general anesthesia on the 11th day after admission. Histopathological examination revealed the lesion as a malignant mixed GCT of the undescended testis composed predominantly of yolk sac tumors with foci of embryonal cell carcinoma and seminoma (Fig. 2a, b). The repeated hematological and tumor markers 2 days after the operation are listed in Table 1. Hemoglobin level was still low; then, 4 Units of suspended red blood cells were transfused to correct anemia with no obvious transfusion reaction. The level of AFP was still high, and the follow-up CT scan showed a gradually enlarged right inguinal mass (Fig. 1c, d) 1 week and 2 weeks after the surgery, respectively. On the 17th day after the operation, the patient underwent right inguinal mass resection under local anesthesia. Pathological examination revealed mixed GCT metastasis (Fig. 2c), accompanied by hemorrhage and necrosis. Subsequently, the patient received salvage chemotherapy with a regimen of cisplatin, etoposide, and ifosfamide. Hematology was closely monitored during the treatment. After the first cycle of chemotherapy, the full blood counts remained within the normal range. CD4 count did not decrease during the course of treatment and remained at 118 cells/ml. Then, the patient was discharged and waited for the next cycle chemotherapy. Unfortunately, on the 4th day after discharge, he showed several symptoms of cancer cachexia including fever, progressive weight loss, pain, and severe weakness. Moreover, a low platelet count, which is a side effect of chemotherapy, was found (20 × 10^9^/L, normal 125–350 × 10^9^/L). Therefore, IL-11 was administered subcutaneously at a dosage of 50 μg/kg/day for thrombocytopenia. Two days later, the platelet count was still low (23 × 10^9^/L); on the next day, the patient died because of severe immunosuppression, a low platelet count and cancer cachexia.

## Discussion

Testicular GCTs are relatively uncommon and account for 1% of male tumors [7]. The incidence of testicular GCTs in China is approximately 1/100,000, accounting for 1–1.5% of male tumors and 3–9% of urinary tumors [8], which are similar results to global statistics [7]. GCTs account for 90–95% of all testicular tumors and include seminomas (35–71%) and NSGCTs [2]. NSGCTs usually have a more invasive course than seminomas [9], and they can be classified as follows: teratoma, embryonal carcinoma, endodermal sinus tumor/yolk sac tumors [10], choriocarcinoma and mixed GCTs. Mixed GCTs are relatively rare in China. A retrospective analysis of the clinical data of 133 patients with testicular tumors at Peking University Third Hospital (Beijing, China) from May 1994 to November 2016 was conducted [11]. The results showed that mixed GCTs accounted for only 16% (22/133) of testicular tumors. To date, no research on HIV-infected GCT patients has been reported in China because of the rarity of this tumor type. EGGCTs account for 1–5% of all GCTs [1] and typically occur at or near the midline [12]. In adults, the most common sites of EGGCTs are the mediastinum, retroperitoneum, and cranium in descending order. Primary retroperitoneal GCTs account for approximately 30% of EGGCTs [9]. Usually, the most general components of mixed GCTs are embryonal carcinoma and teratoma [13], followed by the presence of yolk sac tumors. However, our patient developed a retroperitoneal testicular malignant mixed GCT consisting of yolk sac tumors as the major components and seminoma and embryonal carcinoma as the minor components. As it rarely occurs, the exact incidence of histopathology is unknown. To the best of our knowledge, only two cases have been reported [14, 15]. Furthermore, cryptorchidism is a certain risk factor for testicular cancer [3]. It has been reported that cryptorchidism is associated with a 5- to 10-fold increase in testicular malignancy [16]. Approximately 10% of all cases of GCTs occur in men with a history of cryptorchidism [17]. The relationship between cryptorchidism and GCTs is still unclear. Several explanations have been reported, and one of them indicated that androgen signaling, which is a common regulatory pathway, might be a possible reason for the relationship [18]. Another explanation is that infertility and germ cell tumorigenesis are directly related to the abnormal portion of the testis itself [19].

Acquired immune deficiency syndrome (AIDS)-defining cancers, including non-Hodgkin’s lymphoma, Kaposi’s sarcoma, and invasive cervical cancer, occur more frequently in HIV-infected individuals. However, with the advancement and introduction of cART, the prognosis and survival for patients with HIV have been improved, and the incidence of AIDS-defining cancers has decreased substantially [20–23]. At the same time, the incidence of certain non-AIDS-defining malignancies, such as lung cancer, head and neck cancers, GCTs, anal cancer, hepatocellular carcinoma, and Hodgkin’s lymphoma, has increased significantly, and it has become a considerable factor of mortality in HIV-infected individuals [24, 25]. Compared to the general population, HIV-infected men are 1.4 to 8.2 times more likely to develop testicular tumors [26], particularly seminomas [4]. The mechanisms by which HIV-induced immunodeficiency could increase the risk for GCTs are complex and unclear, and they may involve a combination of factors, including oncogenic viral infection, immunosuppression, impairment of tumor immune surveillance, and an imbalance between cellular proliferation and differentiation [26]. A report showed that two HIV patients developed mixed GCTs subsequent to starting hepatitis C virus (HCV) treatment. In contrast, our patient was not coinfected with HCV. The roles that pegylated-interferon and ribavirin play in carcinogenesis merit further investigation [27].

AFP and β-HCG are serum tumor markers of testicular neoplasia that play important roles in diagnosis, treatment, prognosis, and follow-up [28]. AFP is produced by endodermal sinus tumors, either alone or in association with other types of GCTs, and β-HCG is only produced by syncytiotrophoblasts, which are components of choriocarcinoma. In our case, the serum marker AFP was highly elevated, i.e., 34,222 ng/ml, predominantly indicating the presence of a yolk sac tumor.

The CT performance of primary extragonadal mixed GCTs is nonspecific; these GCTs are depicted as heterogeneous tumors with areas of hemorrhage, necrosis, and heterogeneous enhancement. However, CT can characterize the mass and its relationship to adjacent structures and can identify benign or malignant tumors as well as detect lymph node metastasis and distant metastasis, providing important evidence for tumor staging before treatment and surveillance after therapy.

The diagnosis of GCTs is not very difficult according to the results of serum tumor markers, imaging examinations, and histological evaluations. However, the optimal management of GCTs in HIV-infected patients, especially those with severe immunosuppression, remains uncertain and challenging. There are three possible reasons for the poor outcome of HIV-infected patients with GCTs. First, there are high levels of HIV-related mortality due to opportunistic infections [29]. Second, HIV-infected patients poorly tolerate chemotherapy, resulting in a reduction in the drug dose and patient response rate. Researchers found a similar rate of response to chemotherapy in GCTs between HIV-infected patients and the general population, but they noted that 43% of HIV patients received reduced doses of chemotherapy because of toxicity or poor compliance [30]. Third, due to severe immunosuppression and impairment of tumor immune surveillance, HIV-infected individuals may have a more aggressive clinical course. Our patient presented with bilateral inguinal region metastases and rapid growth of a right inguinal mass, which demonstrated aggressive disease in this HIV patient.

Our patient had a poor outcome and died 1.5 months after exact diagnosis. This case was categorized as stage IIIC and was composed of a variety of germ cell tumor components (including yolk sac tumors, embryonal cell carcinoma and seminoma). The later clinical stage and complicated histological pattern suggested a poor prognosis. Extragonadal primary disease and extremely high levels of AFP are validated prognostic factors of poor disease-free survival [31]. Due to a retroperitoneal primary mass with bilateral inguinal metastases and extremely high levels of AFP (34,222 ng/ml), the patient was classified as poor risk. The initial bulky disease (17 cm × 16 cm × 24 cm) and aggressive nature of the lesion in this HIV-infected individual with severe immunosuppression may be further contributing factors. Patients with retroperitoneal NSGCTs greater than 10 cm in size usually have significantly worse survival rates [32]. Our patient failed to be diagnosed as HIV positive promptly and did not receive cART until admission because of an abdominal mass. Therefore, the patient showed a very low CD4 count of 70 cells/ml, which suggested severe immunosuppression. Another reason for the poor prognosis could be due to delayed and improper management. When the abdominal lump was found in a local hospital and a testicular tumor was suspected, the patient initially refused active treatment at that time. It was not until the tumor had grown rapidly for 3 months and the volume had increases 2–3-fold that the patient returned to the hospital for treatment. Although optimal treatment strategies for NSGCTs in HIV-infected patients have not been established, previous studies have suggested that HIV-infected patients can tolerate standard treatment remarkably well and should be treated with similar strategies used for HIV-negative individuals with GCTs [26, 33–35]. According to the NCCN Guidelines: Testicular Cancer (Version 1.2019), the treatment strategies for NSGCTs among healthy individuals should be based on histology, clinical staging and prognosis evaluations [36]. For poor prognosis and advanced metastatic (stage IIIC) NSGCT patients without HIV infection, standard chemotherapy is the initial treatment option. EGGCT patients are also treated with initial chemotherapy [36]. A BEP (bleomycin, etoposide, and cisplatin) regimen is well tolerated and effective, and it offers good survival and prognosis. Four cycles of BEP with 3-week intervals is the standard [36, 37]. Alternatively, patients who cannot tolerate bleomycin can be treated with 4 cycles of a VIP (etoposide, ifosfamide, and cisplatin) regimen [36, 38]. Studies have demonstrated that conventional-dose chemotherapy is recommended, and first-line high-dose chemotherapy failed to improve the outcome [39, 40]. Enhanced CT scans and serum tumor markers are indicated to assess the response after chemotherapy. If a complete response is found according to the results of imaging and tumor markers, close surveillance is recommended [36]. Patients who experience a partial response to chemotherapy (detection of a residual mass on imaging and/or persistently elevated serum tumor makers) are treated with surgical resection of all residual masses [1, 36, 41]. If only natural teratoma or necrosis is found in the resected tissue, surveillance is recommended. If yolk sac, choriocarcinoma, embryonal, or seminoma elements are encountered in the residual mass, patients should be treated with 2 cycles of chemotherapy [EP (etoposide and cisplatin), TIP (paclitaxel, ifosfamide, and cisplatin), or VIP/VeIP (vinblastine, mesna, ifosfamide, and cisplatin)] [36]. Moreover, one case showed that it was safe to give chemotherapy and HAART simultaneously [26]. Since EGGCTs are rare, HIV-infected patients with EGGCTs are even more rare. Additionally, there is no standardized treatment for HIV-infected EGGCT patients with severe immunosuppression. For the purpose of relieving the patient’s symptoms, radical surgical resection of the retroperitoneal mass and the inguinal metastases was performed, followed by chemotherapy, which was inconsistent with the treatment guidelines for HIV-negative individuals with advanced NSGCTs.

Generally, the outcome of non-HIV-infected extragonadal NSGCT patients is satisfactory. A non-HIV-infected man with unilateral cryptorchidism presented with a rapidly growing mass for 6 months and large (17 cm × 19 cm × 35 cm) mixed GCTs (mostly yolk sac tumors: 90–95%) in the retroperitoneum with elevated levels of AFP (120,000 ng/mL). He then underwent excision of the mass and postoperative chemotherapy. Histology, clinical staging, prognosis evaluations, and treatments were similar in our case, but the patient had a successful short-term survival; a one-year follow-up showed no recurrence, and AFP levels were normalized [15]. Häcker et al. reported a primary retroperitoneal EGGCT patient without HIV infection who received chemotherapy and surgical resection of the residual mass and developed metachronous testicular cancer 10 months later [41]. A prospective trial of chemotherapy in non-HIV-infected patients with EGGCTs demonstrated a significant response in patients with retroperitoneal tumors and a 4-year survival rate of more than 70% [42]. Furthermore, 3-year progression-free survival was achieved in 48 to 54% of HIV-negative patients with EGGCTs by using chemotherapy followed by surgical consolidation [43, 44]. Hashimoto et al. reported that among HIV-negative patients with retroperitoneal NSGCTs, the 5-year survival rate was 94.7% with the use of chemotherapy, and 76.9% of poor-risk patients (10/13) survived for 2 years without evidence of disease [45].

However, the treatment outcome of HIV-positive patients with progressive NSGCTs is uncertain. One HIV-infected patient with advanced mixed GCTs (clinical stage IIC) received chemotherapy at onset but died 1.5 months after tumor diagnosis [46]. A report documented that a BEP regimen offered an initially remarkable response in a cART-naïve HIV patient with bulky abdominal yolk sac tumors and lung metastasis; however, the response did not last, as the patient suffered lung recurrence 5 months after completing chemotherapy [47]. These studies show that NSGCTs in HIV-infected patients were more likely to progress and recur after treatment. Some studies have demonstrated inconsistent results. Fizazi et al. retrospectively analyzed the results of chemotherapy in 34 HIV-infected men with GCTs (18 of them had NSGCTs), and 50% of patients were alive with a median follow-up of 27 months (range, 3–150) [30]. Powles et al. demonstrated that HIV patients with stage II or stage III GCTs treated with standard therapy had similar favorable outcomes compared to the HIV-negative population, and only two of the 14 patients died from the progressive disease at a median follow-up of approximately 4.5 years [26]. Another study found that HIV patients with metastatic GCTs who underwent chemotherapy had a similar disease-free survival compared with the general population [48]. The optimal treatment strategy and therapeutic guidelines for achieving long-term survival in HIV-positive patients with severe immunosuppression and advanced NSGCTs remain to be defined, and more prospective clinical trials should be conducted in the future.

## Conclusions

This case report demonstrated the challenges that pertain to the management of HIV-infected patients with primary EGGCTs due to their aggressive nature, and it evaluated the poor treatment outcome. The standard treatment guidelines for non-HIV-infected GCT patients are important treatment principles for various types of GCTs, but HIV-infected GCT patients with severe immunosuppression need special consideration. The appropriate management of advanced GCTs in HIV-infected patients with severe immunosuppression needs to be established in the future, and collaboration among oncologists, surgeons and HIV physicians is key.

## Abbreviations

AFP: Alpha-fetoprotein
AIDS: Acquired immune deficiency syndrome
cART: Combination antiretroviral therapy
CT: Computed tomography
EGGCTs: Extragonadal germ cell tumors
GCTs: Germ cell tumors
HCV: Hepatitis C virus
HIV: Human immunodeficiency virus
NSGCTs: Nonseminomatous germ cell tumors
β-HCG: Beta-human chorionic gonadotropin
